# Engineering autoreactive T and B cell responses toward active immunotherapy for inflammatory diseases

**DOI:** 10.1073/pnas.2104743118

**Published:** 2021-04-21

**Authors:** Jason Y. H. Chang, Darrell J. Irvine

**Affiliations:** ^a^Koch Institute for Integrative Cancer Research, Massachusetts Institute of Technology, Cambridge, MA 02139;; ^b^Department of Materials Science and Engineering, Massachusetts Institute of Technology, Cambridge, MA 02139;; ^c^Department of Biological Engineering, Massachusetts Institute of Technology, Cambridge, MA 02139;; ^d^Ragon Institute of MGH, MIT and Harvard, Boston, MA 02139;; ^e^Howard Hughes Medical Institute, Chevy Chase, MD 20815

Antiinflammatory therapeutics are commonly used to combat a vast array of chronic inflammatory and autoimmune diseases, including rheumatoid arthritis, inflammatory bowel disease, psoriasis, and Crohn’s disease (1). These chronic inflammatory diseases affect ∼5 to 7% of the population, creating a significant socioeconomic burden and impact on patients’ quality of life (2). Current therapies have revolved around the use of anti-tumor necrosis factor (TNF) antibodies, aiming to block the activity of TNF-α and cytokines such as interleukin (IL)-1, IL-6, and granulocyte-macrophage colony-stimulating factor that make up its downstream proinflammatory cascade (3). Although these anti-TNF therapeutics have shown efficacy over the past two decades, there are several drawbacks to this approach, including the need for repeated injections, patient compliance issues, tolerability, and the development of antidrug antibodies, which could lead to reduction of drug efficacy and adverse side effects such as increased risk of infections and hypersensitivity (4, 5).

**Fig. 1.**
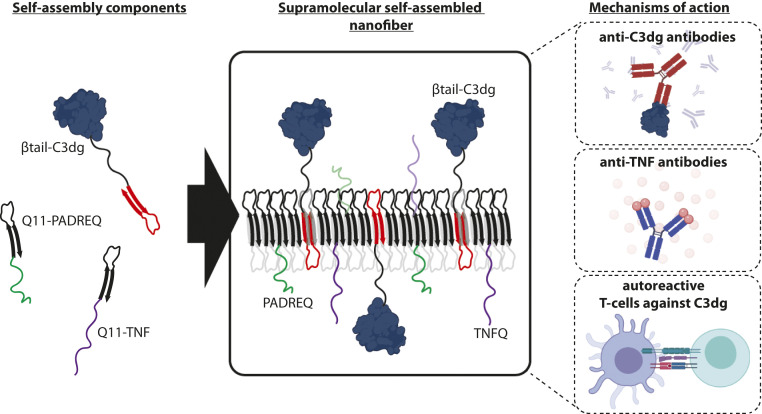
Design of supramolecular nanofibers to modulate inflammation. The C3dg component of complement and short-peptide epitopes derived from the inflammatory cytokine TNF-α were linked to “βtail” peptides, short sequences that self-assemble with themselves in a β-sheet nanoribbon structure, forming long fibers. On injection, these nanofibers are shown to modulate the immune system through three distinct mechanisms: induction of autoreactive antibody responses against endogenous C3dg and TNF as well as priming of antiinflammatory C3dg peptide-specific CD4 T cells.

In PNAS, Hainline et al. (6) developed an alternative active immunotherapy approach that incorporates an engineered fragment of complement protein C3dg and peptide epitopes derived from the soluble form of TNF into a self-assembled supramolecular nanofiber. Administration of these nanofibers as an immunomodulatory vaccine successfully lowered inflammatory signatures in models of TNF-driven septic shock and psoriasis. Despite the simplicity of the components, these nanofibers are shown by the authors to act in multiple complementary ways to modulate the immune system (Fig. 1). First, C3dg acts as a molecular adjuvant to promote B cell activation and costimulation of the complement receptor 2 (CD21) (7), helping to break tolerance against TNF and raise endogenous antibodies against the cytokine. Second, antibodies are directly raised against C3dg, with the potential to limit complement effector functions. Finally, autoreactive helper T cells specific for peptides derived from C3dg are primed. Intriguingly, this last mechanism appears to play an important role in both of the inflammatory models tested here.

Studies have shown that linking C3dg or C3d (a fragment of C3dg) to vaccine immunogens can enhance humoral responses against the antigen, and this is further enhanced through multivalent display of C3dg. Most C3d-adjuvanted vaccine platforms rely on cross-linking C3d to the target antigen to achieve multivalent display, but this approach is random in nature and difficult to control. Alternatively, genetic assembly and expression of recombinant proteins that include C3dg coexpressed with the antigens have also been previously demonstrated, but this approach is often limited in the degree of C3 multimerization that can be achieved (8–11). Hainline et al. (6) elegantly address this problem by incorporating a βtail-tagged C3dg protein and B and T cell peptide epitopes into a supramolecular self-assembly nanofiber platform able to codisplay different proteins in a controlled and modular manner. To highlight the benefits of multivalent display of C3dg-incorparated nanofibers, the authors show increased B cell activation in vitro following treatment with βtail–C3dg nanofibers in a dose-dependent manner, compared to soluble C3dg. This enhanced B cell activation response correlated with stronger antigen-specific antibody titers when mice were immunized with either βtail–C3dg coassembled nanofibers carrying OVAQ (self-assembling ovalbumin peptide epitopes) or βtail–C3dg coassembled nanofibers with TNFQ and PADREQ (B cell epitope peptide and T cell epitope peptide, respectively). Interestingly, these immunizations also induced substantial autoreactive anti-C3dg antibodies, indicating the presence of at least one B cell epitope which proves to work synergistically with the target peptide antigen, as shown by the coassembly of βtail–C3dg, TNFQ, and PADREQ.

To illustrate the antiinflammatory therapeutic properties of this system, Hainline et al. (6) prophylactically immunized mice with different variations of βtail–C3dg and TNFQ coassembled nanofibers then challenged the mice with lipopolysaccharide (LPS) intraperitoneally to induce TNF-mediated inflammation and monitored for shock-like symptoms postchallenge. Here, mice immunized with the coassembled βtail–C3dg/TNFQ/PADREQ nanofibers had complete protection against LPS challenge, whereas 90% of the unimmunized mice developed lethal shock-like symptoms and were removed from the study upon reaching the predetermined cutoffs. Interestingly, mice immunized with only βtail–C3dg or βtail–C3dg/PADREQ nanofibers (without additional TNF antigen) also exhibited therapeutic benefit with reduced inflammation (reaching a 90% survival rate). Splenocytes from mice immunized with the βtail–C3dg/TNFQ combination produced high levels of IL-4 when restimulated with C3dg, indicating that the nanofiber constructs are capable of priming autoreactive C3dg-specific T helper cells in the systemic circulation. Hainline et al. (6) hypothesize that these C3dg-specific autoreactive T helper cells would recognize complement peptides being presented at sites of inflammation, which could trigger these cells to secrete antiinflammatory cytokines or contribute in other regulatory ways to help reduce local or systemic inflammation.

Interestingly, in the LPS challenge model, mice immunized with βtail–C3dg had significantly higher levels of IL-10 in their intraperitoneal lavage and significantly reduced levels of TNF-α in serum and lavage compared to unimmunized mice. These findings suggest an overall synergistic effect of the coassembled βtail–C3dg/TNFQ/PADREQ nanofibers, having both anti-TNF antibody responses as well as anticomplement directed T cell responses. Importantly, the anti-C3dg antibodies raised did not impair the function of the complement cascade or raise an autoimmune response, as the mice were still able to eliminate bacterial infections in a *Listeria monocytogenes* challenge model.

TNF-α also plays a pivotal role in the proinflammatory cascade in psoriasis, where current clinical treatments have revolved around the use of monoclonal anti-TNF antibodies to help reduce local inflammation of the skin. Hainline et al. (6) tested the ability of the coassembled βtail–C3dg/TNFQ/PADREQ nanofibers to reduce local skin inflammation in an imiquimod-induced psoriasis mouse model. Here, mice immunized with the coassembled βtail–C3dg/TNFQ/PADREQ nanofibers exhibited reduced epidermal thickening, comparable to standard-of-care TNF antibody therapy. Upon further investigation of which components of the peptide nanofiber platform contributed to therapeutic efficacy, Hainline et al. (6) vaccinated TNF knockout mice treated with imiquimod and found that animals immunized with βtail–C3dg/TNFQ/PADREQ also exhibited reduced epidermal thickening, indicating that therapeutic efficacy in this model does not relate to anti-TNF humoral responses raised by the nanofibers.

In light of these findings, the authors investigated the therapeutic contributions of anti-C3dg raised in the treated animals by collecting serum from mice immunized with βtail–C3dg and then passively transferring to naïve recipient mice, which were then challenged with LPS. Interestingly, the passively transferred mice had no protection against LPS-induced shock, suggesting that anti-C3dg antibodies also did not play a therapeutic role in the prevention of shock-like symptoms. In fact, in mice immunized with βtail–C3dg nanofibers but depleted of CD4+ T cells prior to LPS challenge, all of the animals succumbed to shock-like symptoms similar to unimmunized mice receiving an isotype control antibody; no protection was observed with an overall survival rate of 0%. Comparatively, control βtail–C3dg-immunized mice exhibited only mild shock-like symptoms with an overall 100% survival rate. Hence, C3dg-specific CD4+ T cells seems to play a pivotal role in reducing local inflammation and preventing endotoxic shock in these animals. Although Hainline et al. (6) suggest that these autoreactive T cells could potentially function as regulatory T cells to moderate effector immune responses and control inflammation, more in-depth T cell characterization will be warranted in the future to help better understand this T cell–dependent protective pathway its consequences on natural and pathological immunity.

Hainline et al. (6) have successfully demonstrated through various applications the benefits of a modular supramolecular coassembly vaccine platform that incorporates C3dg as a molecular adjuvant and TNF-peptide epitopes to help protect against TNF-mediated inflammation. The coassembled nanofibers not only produced strong humoral responses against the target antigen (TNF) through activation of B cells but also elicited C3dg-autoreactive T cells that helped regulate the TNF-mediated inflammatory cascade by mechanisms yet to be defined. While the safety of this approach for human translation will require more study, the system provides an interesting general platform that could be applied to regulate inflammatory states in a variety of disease contexts.
